# Elevated baseline vitamin B12 level and all-cause mortality risk in patients with sepsis: a cohort analysis

**DOI:** 10.3389/fnut.2026.1758059

**Published:** 2026-04-24

**Authors:** Kuo-Chuan Hung, Li-Chen Chang, Jheng-Yan Wu, Yi-Chen Lai, Chun-Ning Ho, Chien-Ming Lin, I-Wen Chen

**Affiliations:** 1 Department of Anesthesiology, Chi Mei Medical Center, Tainan, Taiwan; 2 Department of Anesthesiology, E-Da Hospital, I-Shou University, Kaohsiung, Taiwan; 3 Department of Nutrition, Chi Mei Medical Center, Tainan, Taiwan; 4 Department of Anesthesiology, Chi Mei Medical Center, Liouying, Tainan, Taiwan

**Keywords:** biomarker, cobalamin, mortality, propensity score matching, sepsis, vitamin B12

## Abstract

**Background:**

Elevated serum vitamin B12 levels have been associated with adverse outcomes in various clinical settings; however, the prognostic significance of pre-existing vitamin B12 elevation measured before sepsis onset remains unclear. This study examined the association between pre-sepsis vitamin B12 levels and clinical outcomes in adults with sepsis.

**Methods:**

This retrospective cohort study utilized the TriNetX database to identify adults diagnosed with sepsis between 2010 and 2024. Patients with elevated vitamin B12 levels (≥1,000 pg./mL) measured within 3 months before sepsis diagnosis were compared with those having normal levels (300–900 pg./mL) using 1:1 propensity score matching. The primary outcome was 90-day all-cause mortality. The secondary outcomes included major adverse cardiovascular events, organ failure, intensive care unit (ICU) admission, and progression to severe sepsis.

**Results:**

After propensity score matching, 18,830 patients per group (37,660 patients in total) were included in this cohort. Elevated pre-sepsis vitamin B12 levels were associated with increased 90-day mortality [26.3% vs. 21.8%; hazard ratio (HR): 1.29, 95% confidence interval (CI): 1.24–1.35, *p* < 0.001] and progression to severe sepsis (17.3% vs. 15.3%; HR 1.18, 95% CI 1.12–1.24, *p* < 0.001). Organ failure (HR 1.09, *p* < 0.001) and ICU admission showed a marginal association (HR 1.05, *p* = 0.031), while major adverse cardiovascular events showed no significant association. During the 90-365-day follow-up period, the associations were attenuated (mortality: HR 1.07, 95% CI 0.99–1.15, *p* = 0.077). Dose–response analysis revealed stronger associations at vitamin B12 levels >1,200 pg./mL (HR 1.35 for mortality). Younger patients (18–65 years) exhibited more pronounced associations.

**Conclusion:**

Elevated pre-sepsis vitamin B12 levels are associated with increased short-term mortality and adverse outcomes in patients with sepsis, suggesting that elevated pre-sepsis vitamin B12 levels may serve as a risk-associated biomarker, although its independent prognostic utility requires further validation.

## 1 Introduction

Sepsis remains a major global health challenge, with an estimated 48.9 million cases and 11 million deaths annually, accounting for approximately 20% of all global deaths (1, 2). Despite ongoing advances in early recognition, timely antimicrobial therapy, and modern supportive care strategies, sepsis continues to carry a substantial mortality burden, with reported 90-day mortality rates among affected patients remaining as high as approximately 26.7–32.2% (3–5). Given this considerable disease burden, identifying readily available biomarkers that predict adverse outcomes could facilitate early risk stratification, guide clinical decision-making, and potentially improve survival in this high-risk population (6–8).

Vitamin B12 (cobalamin) is an essential micronutrient traditionally recognized for its role in hematopoiesis and neurological function, with clinical attention historically focused on the consequences of deficiency (9–11). However, accumulating evidence over the past two decades suggests that elevated serum vitamin B12 levels may paradoxically indicate poor prognosis across diverse clinical settings. Salles et al. (12) first demonstrated that high vitamin B12 levels predicted 90-day mortality in elderly inpatients, with a nearly 4.5-fold increased risk even among non-cancer patients. Subsequent studies in medical intensive care unit patients confirmed this association, showing that non-survivors had significantly higher vitamin B12 concentrations than survivors (13). A recent systematic review and dose–response meta-analysis of 22 cohort studies further established that each 100 pmol/L increase in serum vitamin B12 was associated with a 4% higher risk of all-cause mortality in the general population and a 6% higher risk among older adults (14).

High vitamin B12 levels are generally considered a consequence rather than a cause of critical illness, reflecting hepatic dysfunction, tissue injury, or systemic inflammation (15–17). Most prior studies measured vitamin B12 at hospital or intensive care unit (ICU) admission when severe disease is already present, limiting the assessment of whether vitamin B12 elevation precedes illness onset or merely accompanies it. Consequently, the prognostic value of pre-existing elevated vitamin B12, detected before sepsis onset, has not been systematically evaluated. Clarifying this distinction is clinically important because a true pre-illness biomarker could enable earlier risk stratification and intervention prior to clinical deterioration. To address this knowledge gap, we conducted a large-scale retrospective cohort study investigating the relationship between pre-sepsis serum vitamin B12 elevation and mortality among adults with sepsis.

## 2 Methods

### 2.1 Study design and data source

This retrospective cohort study was conducted using the TriNetX research network. The TriNetX platform is a large federated electronic health record network that has been widely utilized in epidemiological and real-world outcome studies across diverse clinical settings (18–22). The database contains information including demographic characteristics, diagnostic codes, procedural records, medication prescriptions, and laboratory measurements. This study aimed to determine whether elevated serum vitamin B12 levels are associated with increased mortality risk in adult patients with sepsis. Approval for this study was obtained from the Institutional Review Board of Chi Mei Medical Center. Given that the research involved secondary analysis of fully de-identified electronic health record data, the requirement for informed consent was waived in accordance with ethical standards governing retrospective research.

### 2.2 Study population and cohort definitions

The study population comprised adults aged 18 years or older with a diagnosis of sepsis (ICD-10-CM code A41) during the observation period (2010–2024). Individuals were classified into two cohorts based on vitamin B12 status measured within 3 months before sepsis diagnosis. The exposure cohort consisted of patients with elevated serum vitamin B12 levels, defined as ≥1,000 pg./mL (High B12 group). The control cohort comprised patients with normal vitamin B12 levels, ranging from 300 pg./mL to 900 pg./mL (13). To avoid misclassification, patients in the exposure cohort who had any vitamin B12 measurement below 1,000 pg./mL within the three-month pre-sepsis window were excluded. Similarly, patients in the control cohort who had any vitamin B12 measurements outside the 300–900 pg./mL range during this period were excluded. The index date for each participant was designated as the date of sepsis diagnosis.

### 2.3 Exclusion criteria

Patients were excluded if they had any of the following conditions prior to the index date: pregnancy; organ transplantation; prior vitamin B12 or vitamin B12/folic acid supplementation; non-infective enteritis and colitis; toxic liver disease; hepatic failure; HIV infection; history of bariatric surgery; end-stage renal disease; CKD stages 4 or 5; dialysis dependence; or malignant neoplasms of lymphoid, hematopoietic, and related tissues.

### 2.4 Propensity score matching

Propensity score matching was performed to balance the baseline characteristics between cohorts. Covariates included demographic factors (age, sex, and race), comorbid conditions (e.g., essential hypertension, alcoholic liver disease, chronic hepatitis, liver fibrosis and cirrhosis, other inflammatory liver diseases), and laboratory parameters (e.g., aspartate aminotransferase, alanine aminotransferase, total bilirubin, and C-reactive protein) (Supplementary Table 1). One-to-one greedy nearest-neighbor matching without replacement was performed using a caliper width of 0.1 standard deviations of the logit of the propensity score. Covariate balance was evaluated using standardized mean differences (SMDs), with values less than 0.1 indicating adequate balance. Propensity score distributions before and after matching were visually inspected and compared to confirm improved overlap and effective reduction of baseline imbalance between the cohorts.

### 2.5 Outcome definitions and follow-up

The primary outcome was all-cause mortality within 90 days of the index date. Secondary outcomes included major adverse cardiovascular events (MACE), defined as the composite of cardiac arrest, acute myocardial infarction, or cerebral infarction; organ failure, defined as the composite of acute kidney failure, acute respiratory failure, acute respiratory distress syndrome, hepatic failure, or disseminated intravascular coagulation; ICU admission; and progression to severe sepsis. Outcomes were assessed during a follow-up window beginning 1 day after the index date and extending to 90 days. To explore short- and long-term effects, all outcomes were additionally evaluated at 30 days and during an extended period between 90 and 365 days after the index date.

### 2.6 Sensitivity analyses

Two sensitivity analyses were performed to assess the robustness of the findings. In Model I, we restricted the exposure window by including only patients whose vitamin B12 level was measured within 6 weeks before sepsis diagnosis, thereby ensuring closer temporal proximity between the laboratory measurement and the index event. In Model II, we restricted the analysis to patients diagnosed with sepsis between 2018 and 2024 to account for potential temporal changes in clinical practice patterns, coding practices, and sepsis management protocols during the study period.

### 2.7 Dose–response analysis

To evaluate potential dose–response relationships between vitamin B12 status and adverse outcomes, an additional analysis was conducted comparing patients with markedly elevated vitamin B12 levels (>1,200 pg./mL) against the control cohort with normal vitamin B12 levels (300–900 pg./mL). The same propensity score matching approach and outcome evaluation strategy were applied during the 90-day follow-up period. This study aimed to determine whether a graded association exists between increasing vitamin B12 levels and mortality risk.

### 2.8 Statistical analysis

The database provides de-identified, aggregated patient-level information, and individual-level data cannot be accessed or exported. All data management procedures are performed by the contributing institutions within the network. Statistical analyses were conducted by the study investigators using the built-in analytics tools of the TriNetX platform. The authors had full responsibility for study design, data analysis, interpretation of the results, and manuscript preparation. Time-to-event analyses were conducted using Kaplan–Meier methods to estimate the cumulative event incidence over the pre-specified follow-up intervals, with the index date designated as time zero. Participants were censored at the earliest occurrence of death, last documented clinical encounter, or completion of the observation period. Group differences in survival curves were compared using the log-rank test. Kaplan–Meier survival curves were generated using Python (matplotlib library, version 3.5.0). Survival probabilities with corresponding 95% confidence intervals were plotted over time to visualize between-group differences. Associations between exposure and outcomes were quantified using Cox proportional hazards regression models, yielding hazard ratios (HRs) and corresponding 95% confidence intervals (CIs). The proportional hazards assumption was evaluated through the assessment of Schoenfeld residuals and formal testing for time-dependent covariate effects.

Subgroup analyses were performed according to sex (male versus female) and age (18–65 years versus >65 years). Potential effect modification was examined by incorporating interaction terms into the regression models, with interaction *p*-values reported to determine whether the association between elevated vitamin B12 levels and mortality varied across subgroups. All survival analyses were conducted in propensity score–matched cohorts to minimize baseline confounding. Statistical significance was defined as a two-sided p-value <0.05.

## 3 Results

### 3.1 Patient selection and baseline characteristics

**Figure 1:**
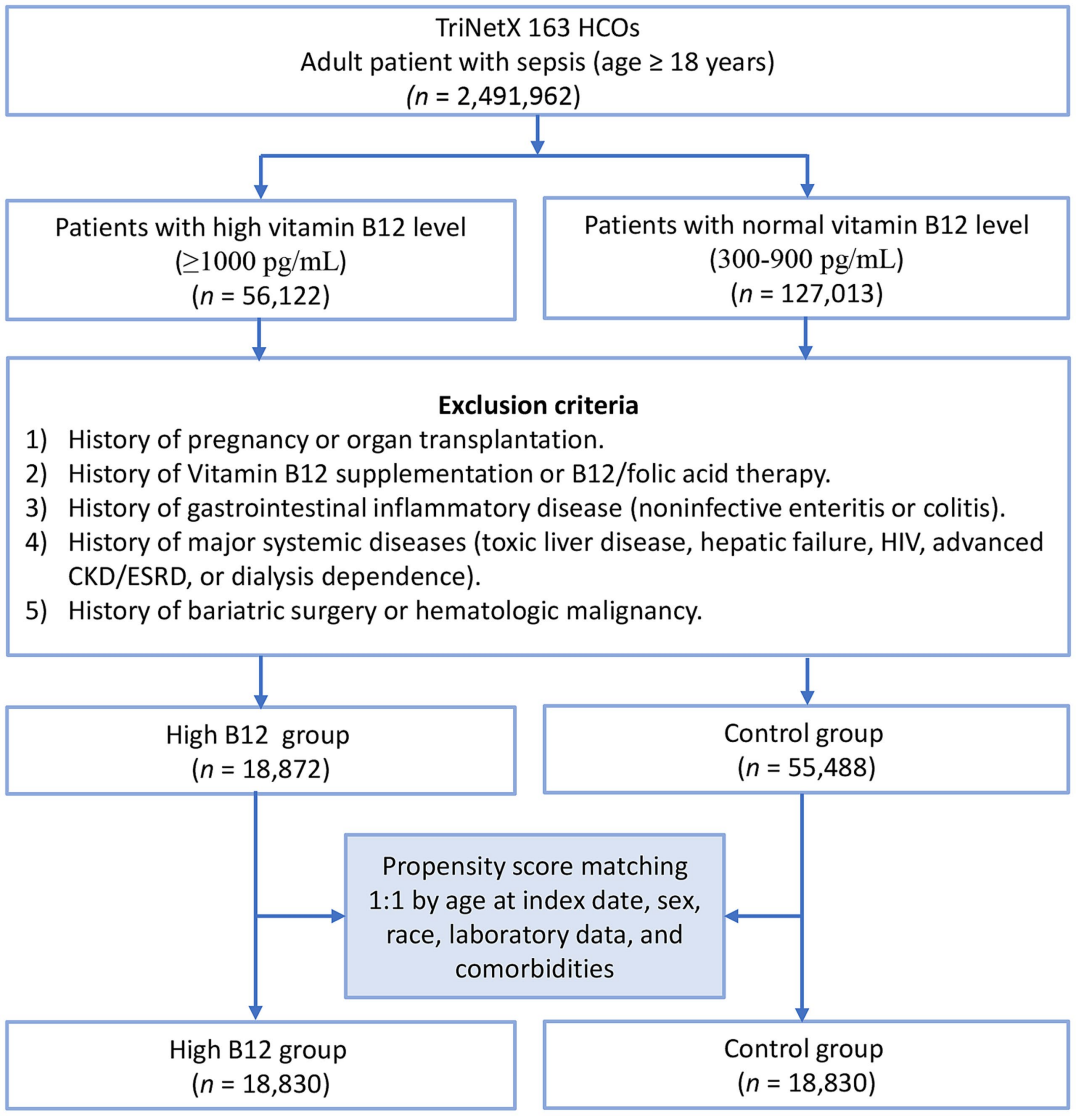
Patient selection flowchart from the TriNetX database. HCOs, Healthcare Organizations; CKD, chronic kidney disease; HIV, human immune virus; ESRD, end-stage renal disease; High B12, high vitamin B12 level.

**Figure 2:**
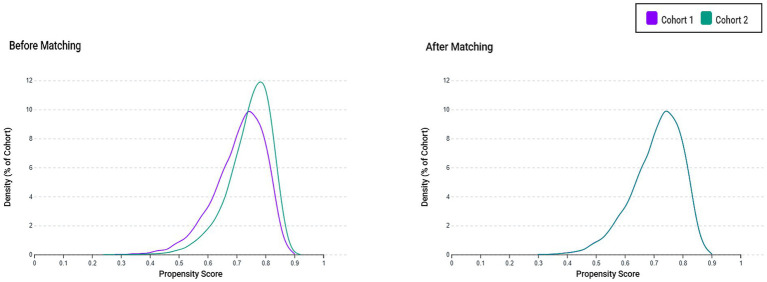
Propensity score density distributions before and after matching. Density plots illustrate the distribution of propensity scores for the high vitamin B12 group (Cohort 1) and the control group (Cohort 2), demonstrating a substantial baseline imbalance before matching and an improved covariate balance after propensity score matching.

The initial query of the TriNetX database identified 18,872 patients with elevated serum vitamin B12 levels (≥1,000 pg./mL) and 55,488 patients with normal vitamin B12 levels (300–900 pg./mL) measured within 3 months before sepsis diagnosis (Figure 1). Before propensity score matching, notable baseline imbalances were observed between cohorts, with standardized mean differences exceeding 0.1 for several variables including acute kidney failure (45.3% vs. 38.9%, SMD = 0.131), acute respiratory failure (33.5% vs. 25.7%, SMD = 0.173), malnutrition (26.5% vs. 18.1%, SMD = 0.204), albumin levels (65.0% vs. 72.9%, SMD = 0.171), and hemoglobin (SMD = 0.140). Following one-to-one propensity score matching, 18,830 patients remained in each cohort, with adequate balance achieved across all covariates (SMD < 0.1) (Table 1). The matched cohorts had comparable demographic profiles, with a mean age of 69.0 ± 15.9 years, approximately half of the patients being female, and white race accounting for about 62% in both groups. Propensity score density distributions showed substantial overlap after matching, confirming the effective reduction of baseline confounding (Figure 2).

**Table 1:** Baseline characteristics of patients with sepsis before and after propensity score matching.

Variables	Before matching			After matching		
	High B12 group (*n* = 18,872)	Control group (*n* = 55,488)	SMD^a^	High B12 group (*n* = 18,830)	Control group (*n* = 18,830)	SMD^a^
Patient characteristics						
Age at index (years)	69.0 ± 15.9	68.9 ± 16.0	0.006	69.0 ± 15.9	69.0 ± 15.9	<0.001
Female	9,689 (51.3)	26,301 (47.4)	0.079	9,663 (51.3)	9,759 (51.8)	0.010
BMI ≥ 30 kg/m^2^	5,586 (29.6)	18,459 (33.3)	0.079	5,584 (29.7)	5,596 (29.7)	0.001
White	11,675 (61.9)	36,604 (66.0)	0.086	11,662 (61.9)	11,714 (62.2)	0.006
Black or African American	3,161 (16.8)	7,575 (13.7)	0.086	3,145 (16.7)	3,106 (16.5)	0.006
Unknown Race	2,143 (11.4)	7,029 (12.7)	0.040	2,143 (11.4)	2,213 (11.8)	0.012
Asian	1,080 (5.7)	2078 (3.7)	0.093	1,070 (5.7)	1,015 (5.4)	0.013
Comorbidities						
Essential (primary) hypertension	11,853 (62.8)	36,461 (65.7)	0.061	11,831 (62.8)	11,787 (62.6)	0.005
Dyslipidemia	9,258 (49.1)	28,787 (51.9)	0.056	9,242 (49.1)	9,218 (49.0)	0.003
Acute kidney failure	8,557 (45.3)	21,575 (38.9)	0.131	8,525 (45.3)	8,503 (45.2)	0.002
Neoplasms	7,127 (37.8)	19,231 (34.7)	0.065	7,103 (37.7)	7,114 (37.8)	0.001
Ischemic heart diseases	6,791 (36.0)	19,900 (35.9)	0.003	6,779 (36.0)	6,752 (35.9)	0.003
Acute respiratory failure	6,326 (33.5)	14,238 (25.7)	0.173	6,295 (33.4)	6,326 (33.6)	0.003
Heart failure	5,667 (30.0)	16,090 (29.0)	0.023	5,656 (30.0)	5,661 (30.1)	0.001
Malnutrition	5,003 (26.5)	10,025 (18.1)	0.204	4,971 (26.4)	5,003 (26.6)	0.004
Chronic kidney disease (CKD)	4,809 (25.5)	13,759 (24.8)	0.016	4,798 (25.5)	4,782 (25.4)	0.002
Disorders of thyroid gland	4,397 (23.3)	12,370 (22.3)	0.024	4,385 (23.3)	4,383 (23.3)	<0.001
Overweight and obesity	3,922 (20.8)	13,374 (24.1)	0.080	3,921 (20.8)	3,910 (20.8)	0.001
COPD	3,695 (19.6)	12,081 (21.8)	0.054	3,689 (19.6)	3,702 (19.7)	0.002
Nicotine dependence	3,009 (15.9)	10,405 (18.8)	0.074	3,002 (15.9)	3,022 (16.0)	0.003
Dementia	2,449 (13.0)	7,499 (13.5)	0.016	2,446 (13.0)	2,397 (12.7)	0.008
Vitamin D deficiency	2,268 (12.0)	8,111 (14.6)	0.077	2,264 (12.0)	2,279 (12.1)	0.002
Alcohol related disorders	1,112 (5.9)	3,124 (5.6)	0.011	1,095 (5.8)	1,116 (5.9)	0.005
Fibrosis and cirrhosis of liver	876 (4.6)	1,632 (2.9)	0.089	853 (4.5)	846 (4.5)	0.002
Systemic connective tissue disorders	635 (3.4)	1925 (3.5)	0.006	632 (3.4)	663 (3.5)	0.009
Other inflammatory liver diseases	510 (2.7)	999 (1.8)	0.061	502 (2.7)	505 (2.7)	0.001
Alcoholic liver disease	378 (2.0)	539 (1.0)	0.085	357 (1.9)	357 (1.9)	<0.001
Chronic hepatitis	29 (0.2)	85 (0.2)	0.000	29 (0.2)	31 (0.2)	0.003
Laboratory data						
Albumin ≥3.5 g/dL	12,266 (65.0)	40,444 (72.9)	0.171	12,255 (65.1)	12,265 (65.1)	0.001
eGFR ≥60 mL/min/1.73m^2^	15,147 (80.3)	44,247 (79.7)	0.013	15,110 (80.2)	15,073 (80.0)	0.005
Hemoglobin A1c ≥ 7%	3,525 (18.7)	11,036 (19.9)	0.031	3,521 (18.7)	3,485 (18.5)	0.005
Hemoglobin≥ 12 g/dL	12,699 (67.3)	40,871 (73.7)	0.140	12,685 (67.4)	12,624 (67.0)	0.007
ALT (7–55 U/L)	17,569 (93.1)	52,094 (93.9)	0.032	17,532 (93.1)	17,573 (93.3)	0.009
AST (8–48 U/L)	16,348 (86.6)	48,979 (88.3)	0.050	16,318 (86.7)	16,388 (87.0)	0.011
Total bilirubin (0.3–1.2 mg/dL)	16,307 (86.4)	48,271 (87.0)	0.017	16,276 (86.4)	16,302 (86.6)	0.004
C-reactive protein ≥ 10 mg/L	7,113 (37.7)	19,723 (35.5)	0.045	7,096 (37.7)	7,210 (38.3)	0.012

Data are presented as mean ± standard deviation for continuous variables and *n* (%) for categorical variables. High B12, high vitamin B12 level; BMI, body mass index; SMD, standardized mean difference; eGFR, estimated glomerular filtration rate.

a SMD values <0.1 indicate adequate balance between groups after matching.

### 3.2 Outcomes at 90-day follow-up

**Figure 3:**
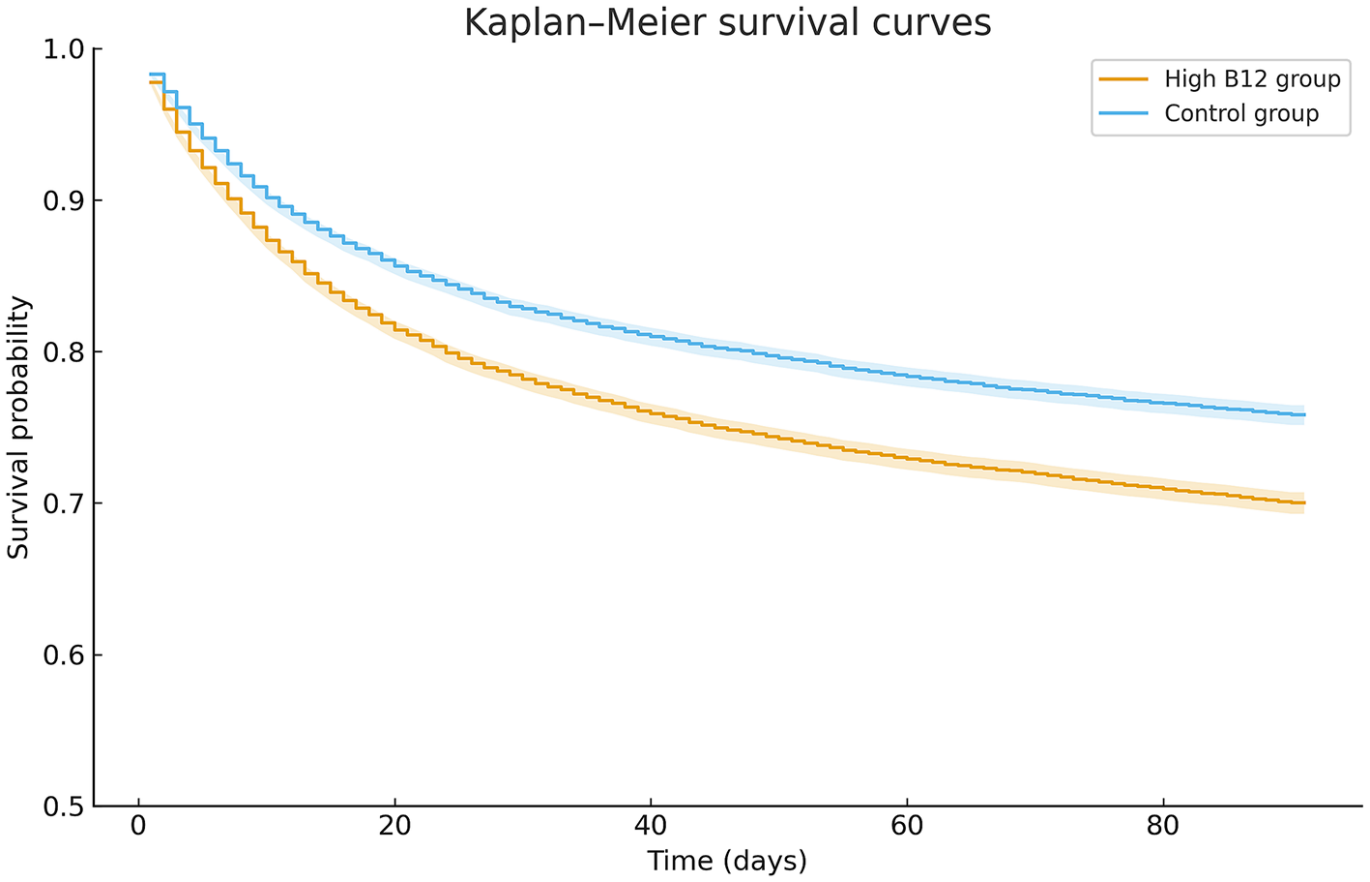
Kaplan–Meier survival curves for 90-day all-cause mortality according to pre-sepsis vitamin B12 levels. The figure depicts Kaplan–Meier survival estimates for patients with elevated serum vitamin B12 levels (high B12 group) and those with normal vitamin B12 levels (control group). Step curves represent the estimated survival probabilities over time, and shaded bands indicate the corresponding 95% confidence intervals. The *y*-axis is truncated at 0.5 to better visualize between-group differences in survival.

During the 90-day follow-up period, elevated pre-sepsis vitamin B12 levels were associated with increased all-cause mortality, with 4,952 deaths (26.3%) in the high B12 group compared to 4,102 deaths (21.8%) in the control group (HR 1.29, 95% CI 1.24–1.35, *p* < 0.001) (Table 2). Kaplan–Meier survival curves demonstrated progressively divergent survival probabilities between the groups throughout the observation period (Figure 3). The high B12 cohort also demonstrated higher rates of organ failure (35.1% vs. 33.8%; HR 1.09, 95% CI 1.05–1.12, *p* < 0.001) and progression to severe sepsis (17.3% vs. 15.3%; HR 1.18, 95% CI 1.12–1.24, p < 0.001). ICU admission rates were marginally higher in the high B12 group (18.4% vs. 18.0%; HR 1.05, 95% CI 1.00–1.10, *p* = 0.031). However, no significant difference was observed in MACEs between cohorts (11.0% vs. 11.3%; HR 1.02, 95% CI 0.96–1.08, *p* = 0.595).

**Table 2:** Association between elevated pre-sepsis vitamin B12 levels and clinical outcomes at 90-day follow-up.

Outcomes	High B12 group^a^ events (%)	Control group^a^ events (%)	HR (95% CI)	*p*-value
Mortality	4,952 (26.3%)	4,102 (21.8%)	1.29 (1.24–1.35)	<0.001
MACE	2,080 (11.0%)	2,120 (11.3%)	1.02 (0.96–1.08)	0.595
Organ failure	6,614 (35.1%)	6,359 (33.8%)	1.09 (1.05–1.12)	<0.001
ICU admission	3,468 (18.4%)	3,386 (18.0%)	1.05 (1.00–1.10)	0.031
Severe sepsis	3,265 (17.3%)	2,876 (15.3%)	1.18 (1.12–1.24)	<0.001

Data are presented as the number of events (percentage). High B12, high vitamin B12 level; HR, hazard ratio; CI, confidence interval; MACE, major adverse cardiovascular events; ICU, intensive care unit.

a *n* = 18,830 for each group after propensity score matching.

### 3.3 Outcomes at short and long-term follow-up

Analysis stratified by temporal intervals revealed that associations between elevated vitamin B12 and adverse outcomes were most pronounced during the initial 30-day period, with mortality demonstrating a hazard ratio of 1.31 (95% CI 1.25–1.37, *p* < 0.001) (Table 3). Similar patterns were observed for organ failure (HR 1.08, 95% CI 1.04–1.12, *p* < 0.001) and severe sepsis (HR 1.18, 95% CI 1.12–1.25, *p* < 0.001). During the extended follow-up period from 90 to 365 days, most associations were attenuated and no longer reached statistical significance, with mortality showing a hazard ratio of 1.07 (95% CI 0.99–1.15, *p* = 0.077). The association with severe sepsis persisted, but was substantially weakened during the later period (HR 1.12, 95% CI 1.00–1.26, *p* = 0.044).

**Table 3:** Temporal associations between elevated pre-sepsis vitamin B12 levels and clinical outcomes at 30-day and 90–365-day follow-up.

Outcomes	30-day^a^		90–365 day^a^	
	HR (95% CI)	*p*-value	HR (95% CI)	*p*-value
Mortality	1.31 (1.25–1.37)	<0.001	1.07 (0.99–1.15)	0.077
MACE	1.00 (0.94–1.07)	0.976	0.99 (0.90–1.09)	0.855
Organ failure	1.08 (1.04–1.12)	<0.001	0.99 (0.93–1.05)	0.708
ICU admission	1.06 (1.00–1.11)	0.028	1.02 (0.93–1.12)	0.618
Severe sepsis	1.18 (1.12–1.25)	<0.001	1.12 (1.00–1.26)	0.044

HR, hazard ratio; CI, confidence interval; High B12, high vitamin B12 level; MACE, major adverse cardiovascular events; ICU, intensive care unit.

a *n* = 18,830 for each group after propensity score matching.

### 3.4 Sensitivity analysis

Two sensitivity analyses confirmed the robustness of the primary findings (Table 4). Model I, restricting the exposure window to 6 weeks before sepsis diagnosis (n = 15,594 per group), demonstrated consistent associations with mortality (HR 1.32, 95% CI 1.26–1.38, *p* < 0.001), organ failure (HR 1.08, 95% CI 1.04–1.12, p < 0.001), and severe sepsis (HR 1.17, 95% CI 1.11–1.24, *p* < 0.001). Model II, limiting the analysis to sepsis cases diagnosed between 2018 and 2024 (*n* = 14,626 per group), yielded similar results for mortality (HR 1.31, 95% CI 1.25–1.37, *p* < 0.001).

**Table 4:** Sensitivity analyses examining the association between elevated pre-sepsis vitamin B12 levels and clinical outcomes.

Outcomes	Model I (*n* = 15,594)		Model II (*n* = 14,626)	
	HR (95% CI)	*p*-value	HR (95% CI)	*p*-value
Mortality	1.32 (1.26–1.38)	<0.001	1.31 (1.25–1.37)	<0.001
MACE	0.99 (0.92–1.05)	0.663	0.97 (0.91–1.04)	0.440
Organ failure	1.08 (1.04–1.12)	<0.001	1.06 (1.02–1.10)	<0.001
ICU admission	1.07 (1.02–1.13)	0.011	1.06 (1.00–1.12)	0.030
Severe sepsis	1.17 (1.11–1.24)	<0.001	1.11 (1.05–1.17)	<0.001

HR, hazard ratio; CI, confidence interval; MACE, major adverse cardiovascular events; ICU, intensive care unit. Model I restricted the exposure window to B12 measurements within 6 weeks before sepsis diagnosis (*n* = 15,594 per group). Model II restricted the analysis to sepsis cases diagnosed between 2018 and 2024 (*n* = 14,626 per group).

### 3.5 Dose–response analysis

Patients with markedly elevated vitamin B12 levels (>1,200 pg./mL, *n* = 12,417 per group) demonstrated a stronger association with 90-day mortality compared with the primary analysis, with a HR of 1.35 (95% CI 1.28–1.42, *p* < 0.001) (Table 5). This cohort also showed associations with organ failure (HR 1.05, 95% CI 1.01–1.10, *p* = 0.017) and severe sepsis (HR 1.20, 95% CI 1.13–1.28, *p* < 0.001). During the 90–365 day follow-up period, the associations with mortality, organ failure, and severe sepsis were not sustained. Conversely, a lower risk of MACE was observed in patients with markedly elevated vitamin B12 levels (HR 0.87, 95% CI 0.78–0.98, *p* = 0.020).

**Table 5:** Association between markedly elevated pre-sepsis vitamin B12 levels (>1,200 pg./mL) and clinical outcomes at 90-day and 90–365-day follow-up.

Outcomes	90 day^a^		90–365 day^a^	
	HR (95% CI)	*p*-value	HR (95% CI)	*p*-value
Mortality	1.35 (1.28–1.42)	<0.001	0.96 (0.87–1.05)	0.338
MACE	0.97 (0.90–1.05)	0.450	0.87 (0.78–0.98)	0.020
Organ failure	1.05 (1.01–1.10)	0.017	0.97 (0.90–1.05)	0.426
ICU admission	1.03 (0.98–1.10)	0.252	0.99 (0.89–1.12)	0.914
Severe sepsis	1.20 (1.13–1.28)	<0.001	1.08 (0.94–1.23)	0.305

HR, hazard ratio; CI, confidence interval; MACE, major adverse cardiovascular events; ICU, intensive care unit. Patients with markedly elevated vitamin B12 levels (>1,200 pg/mL) were compared with the control cohort with normal vitamin B12 levels (300–900 pg/mL).

a *n* = 12,417 for each group after propensity score matching.

### 3.6 Subgroup analysis

Subgroup analyses by sex showed broadly similar associations in men and women, with hazard ratios for mortality of 1.34 (95% CI 1.26–1.42) in males and 1.30 (95% CI 1.22–1.38) in females (Table 6). A significant interaction was observed for ICU admission (*p* = 0.008), with males demonstrating stronger associations than females. Age-stratified analysis showed differential effects, with younger patients (18–65 years) exhibiting more pronounced associations with mortality (HR 1.44) than older patients (HR 1.27, interaction *p* = 0.034) (Table 7). Similar age-related interactions were observed for organ failure (interaction, *p* = 0.001) and severe sepsis (interaction, *p* = 0.002).

**Table 6:** Sex-stratified analysis of the association between elevated pre-sepsis vitamin B12 levels and clinical outcomes at 90-day follow-up.

Outcomes	Male (*n* = 9,162)		Female (*n* = 9,645)		P for interaction
	HR (95% CI)	*p*-value	HR (95% CI)	*p*-value	
Mortality	1.34 (1.26–1.42)	<0.001	1.30 (1.22–1.38)	<0.001	0.488
MACE	0.98 (0.90–1.07)	0.623	1.05 (0.96–1.14)	0.271	0.268
Organ failure	1.08 (1.03–1.13)	0.001	1.05 (1.00–1.10)	0.055	0.406
ICU admission	1.16 (1.09–1.24)	<0.001	1.02 (0.96–1.10)	0.511	0.008
Severe sepsis	1.19 (1.11–1.28)	<0.001	1.13 (1.05–1.22)	0.001	0.328

HR, hazard ratio; CI, confidence interval; MACE, major adverse cardiovascular events; ICU, intensive care unit.

**Table 7:** Age-stratified analysis of the association between elevated pre-sepsis vitamin B12 levels and clinical outcomes at 90-day follow-up.

Outcomes	18–65 (*n* = 4,669)		>65 (*n* = 14,147)		P for interaction
	HR (95% CI)	*p*-value	HR (95% CI)	*p*-value	
Mortality	1.44 (1.30–1.59)	<0.001	1.27 (1.21–1.33)	<0.001	0.034
MACE	1.05 (0.92–1.21)	0.445	1.00 (0.94–1.07)	0.638	0.537
Organ failure	1.22 (1.13–1.31)	<0.001	1.05 (1.01–1.09)	0.012	0.001
ICU admission	1.06 (0.96–1.17)	0.221	1.06 (0.99–1.12)	0.050	1.000
Severe sepsis	1.38 (1.24–1.53)	<0.001	1.13 (1.07–1.20)	<0.001	0.002

HR, hazard ratio; CI, confidence interval; MACE, major adverse cardiovascular events; ICU, intensive care unit.

## 4 Discussion

This large-scale retrospective cohort study revealed that elevated vitamin B12 levels (≥1,000 pg./mL) measured within 3 months before sepsis diagnosis were associated with increased 90-day all-cause mortality (HR 1.29) and progression to severe sepsis (HR 1.18). These associations were most pronounced during the initial 30 days and demonstrated dose–response characteristics, with markedly elevated vitamin B12 levels (>1,200 pg./mL) showing stronger associations with mortality (HR, 1.35). Subgroup analyses revealed differential effects across age categories, with younger patients exhibiting more pronounced associations. While the risk of organ failure (HR 1.09) and ICU admission rates (HR 1.05) were statistically higher in the high B12 group, the minimal effect size suggests limited clinical significance. MACEs were not significantly associated with vitamin B12 status.

Cumulative evidence suggests that the mechanisms linking elevated vitamin B12 to adverse outcomes likely involve hepatocellular injury with subsequent release of intracellular B12 and B12-binding proteins into circulation, indicating compromised hepatic synthetic function and detoxification capacity. The liver’s central role in both vitamin B12 storage and acute phase protein synthesis makes hepatic dysfunction a plausible common pathway connecting elevated vitamin B12 levels to poor outcomes (23, 24). Additionally, elevated serum vitamin B12 levels are frequently observed in dysimmune and chronic inflammatory conditions and are thought to reflect the altered production of cobalamin-binding proteins by activated leukocytes, suggesting that a high vitamin B12 status in this setting may indicate pre-existing immune dysregulation (17, 25, 26). In our cohort, the paradoxical coexistence of elevated serum vitamin B12 levels with higher rates of malnutrition and lower albumin levels supports the interpretation that a high vitamin B12 status reflects underlying metabolic or inflammatory dysregulation rather than true nutritional sufficiency. This apparent contradiction likely represents impaired cellular vitamin B12 uptake despite high circulating levels, creating a functional deficiency at the tissue level that compromises mitochondrial energy metabolism and nucleotide synthesis during the heightened metabolic demands of critical illnesses, which may be associated with organ dysfunction and increased mortality risk.

The observed 29% increased hazard for 90-day mortality associated with elevated pre-sepsis vitamin B12 levels aligns with the results of previous studies. Sviri et al. (13) reported that mean serum vitamin B12 levels were markedly higher in ICU non-survivors than in survivors, and elevated vitamin B12 levels (>900 pg./mL) remained independently associated with increased 90-day mortality. A systematic review by Liu et al. synthesized evidence from 22 cohort studies demonstrating that each 100 pmol/L increment in serum vitamin B12 was associated with 4% increased all-cause mortality in the general population and 6% increased risk among older adults (14). However, the meta-analysis (14) predominantly included stable outpatient cohorts with baseline vitamin B12 measurements and long-term follow-up (3.4–15.7 years), without assessing pre–acute illness vitamin B12 levels or short-term outcomes such as 90-day mortality. This temporal distinction is crucial: their long-term follow-up captured chronic disease progression and gradual physiological decline (14), whereas our study specifically evaluated whether elevated vitamin B12 measured before sepsis diagnosis predicts acute decompensation during the critical early phase of sepsis. This temporal specification supports the possibility that elevated vitamin B12 levels may reflect pre-existing physiological vulnerability, rather than solely representing an acute phase response. The current study addresses this knowledge gap by examining pre-sepsis vitamin B12 levels and their association with short-term outcomes in a critically ill population.

The observed dose–response relationship provides additional evidence supporting an association between higher vitamin B12 levels and adverse outcomes. Across higher concentration strata, HRs increased stepwise (approximately 1.29 at levels ≥1,000 pg./mL, rising to ~1.35 at levels exceeding 1,200 pg./mL), fulfilling the Bradford Hill criterion (27) of a biological gradient and suggesting clinically relevant thresholds for risk stratification. Consistent dose-dependent associations between increasing vitamin B12 levels and mortality have also been reported in several large population-based cohort studies (28, 29) and a recent dose–response meta-analysis (14). The robustness of the findings was confirmed through sensitivity analyses restricting both the temporal window of vitamin B12 measurement to 6 weeks and the diagnostic period to 2018–2024, yielding consistent effect estimates across different analytical approaches.

During the 90–365-day follow-up, the mortality risk approximated unity (HR 1.07, *p* = 0.077), indicating that elevated pre-sepsis vitamin B12 levels are more strongly associated with short-term than prolonged long-term mortality risk. This temporal pattern may be explained by several mechanisms. First, elevated vitamin B12 levels may reflect underlying physiological vulnerability, including hepatic dysfunction, systemic inflammation, and immune dysregulation, which are particularly relevant during the early, high-acuity phase of sepsis and may predispose patients to early mortality. Second, patients who survive beyond the initial 90-day period may represent a selected subgroup with greater physiological reserve, leading to a survivor (selection) effect that attenuates the observed association in later follow-up. Third, early mortality in sepsis is predominantly driven by acute infection-related complications and organ failure, whereas longer-term mortality is more influenced by chronic comorbidities, recovery trajectories, and post-sepsis syndrome, which may dilute the relative contribution of baseline vitamin B12 status. Therefore, patients surviving the initial 90-day period likely represent a selected cohort with sufficient physiological reserve to overcome acute sepsis challenges, potentially diminishing the prognostic relevance of baseline vitamin B12 status. The observed lower risk of MACE in the high B12 group during the long-term follow-up (in the dose–response analysis) may reflect a survivor bias, where the highest-risk patients succumbed early, leaving a survivor cohort with paradoxically lower cardiovascular risk. These findings indicate that vitamin B12 measurement may be most valuable for identifying patients at risk of early complications rather than predicting extended outcomes in this patient population.

The absence of a significant association between elevated vitamin B12 levels and major adverse cardiovascular events contrasts with the study by Wolffenbuttel et al. (30), which reported increased cardiovascular mortality in the general population. The intense inflammatory and hemodynamic stress of sepsis may attenuate the detection of more modest cardiovascular risk signals associated with elevated vitamin B12 in stable populations. During the early post-sepsis period, infection-related complications dominate clinical outcomes, and competing mortality risks may further obscure cardiovascular event ascertainments. These observations suggest that elevated vitamin B12 levels may exhibit outcome-specific rather than uniform prognostic associations in sepsis. Therefore, evaluating these relationships according to follow-up duration and outcome type is important for defining the prognostic significance of vitamin B12 levels in critically ill populations.

In the current study, younger patients (18–65 years) demonstrated substantially stronger associations for mortality (HR 1.44 vs. 1.27, interaction *p* = 0.034), organ failure, and severe sepsis compared with older patients. Elevated vitamin B12 in younger individuals may represent a more pathologically significant deviation from physiological norms, as age-related vitamin B12 increases occur in older populations due to declining renal clearance and altered vitamin B12-binding protein metabolism. Consequently, elevated vitamin B12 levels in younger patients may more strongly indicate hepatic dysfunction, occult hematological disorders, or severe malnutrition that substantially compromises sepsis resilience. Alternatively, older patients’ higher baseline comorbidity burden may introduce competing risk factors that dilute the relative contribution of elevated vitamin B12 levels to adverse outcomes.

For clinical implications, elevated vitamin B12 may serve as a non-specific marker associated with increased risk, potentially facilitating earlier identification of patients who may require closer monitoring. However, it should not be interpreted as an independent or definitive prognostic parameter, as the observed associations may be influenced by residual confounding factors, including infection subtype, severity of organ dysfunction, and underlying comorbidity burden. Therefore, vitamin B12 should be considered as a complementary indicator within a broader clinical context rather than a standalone decision-making tool. In addition, the specificity of vitamin B12 for particular outcome domains, notably the lack of association with cardiovascular events, suggests that vitamin B12 may be considered alongside other clinical indicators, although its incremental value remains uncertain. Prospective validation studies are necessary to determine whether knowledge of elevated vitamin B12 levels influences management decisions and improves outcomes beyond the current standard approaches.

This study had several important limitations. First, the retrospective design precludes causal inference, and unmeasured confounding remains possible despite propensity score matching. Importantly, several clinically relevant factors that may influence sepsis outcomes were not fully captured in the present analysis. These include the type of infection (e.g., bacterial, viral, or fungal), severity and trajectory of organ dysfunction, pathogen virulence, and treatment-related variables such as antimicrobial timing and source control. These factors may independently affect mortality risk and could partially account for the observed association between elevated vitamin B12 levels and adverse outcomes. Second, laboratory vitamin B12 measurements were obtained during routine clinical care rather than using standardized protocols, potentially introducing measurement variability and selection bias toward patients with clinical indications for testing. Third, the definition of elevated vitamin B12 (≥1,000 pg./mL) lacks universal standardization, and assay methods may vary across healthcare organizations. Fourth, the study could not capture vitamin B12 level changes during follow-up or the clinical interventions implemented in response to elevated vitamin B12 levels. Fifth, the exclusion criteria may have selected a population with distinct characteristics, potentially limiting generalizability. Finally, the mechanisms underlying elevated pre-sepsis vitamin B12 levels could not be definitively determined from the available data.

## 5 Conclusion

Elevated serum vitamin B12 levels measured before sepsis diagnosis are associated with increased short-term mortality and progression to severe sepsis, with stronger associations in younger patients. These findings suggest that pre-sepsis vitamin B12 status may serve as a risk-associated biomarker for early risk stratification, although their independent prognostic value requires further validation in prospective studies. Future prospective studies should investigate the mechanisms linking elevated vitamin B12 to adverse sepsis outcomes, evaluate whether vitamin B12 measurement improves existing risk prediction models, and determine whether enhanced monitoring and targeted interventions in high-risk patients identified through vitamin B12 screening can improve clinical outcomes.

## Data Availability

The raw data supporting the conclusions of this article will be made available by the authors, without undue reservation.
