# Supplementary material for: Elevated baseline vitamin B12 level and all-cause mortality risk in patients with sepsis: a cohort analysis
Source: Front Nutr. 2026 Apr 24;13:1758059. doi: 10.3389/fnut.2026.1758059 (PMC13152825; doi:10.3389/fnut.2026.1758059)
Supplement: Supplementary file 1 [file Table_1.docx]

| **Supplemental table 1. Summary of Exposure, Outcomes, Matching Variables, and Exclusion Codes** | | |
| --- | --- | --- |
| Variable / Outcome | Code system | Code |
| Exposure / Index definition | | |
| Sepsis diagnosis | ICD-10-CM (UMLS) | A41 |
| Serum vitamin B12 test | TriNetX laboratory | TNX:9065 |
| Outcomes |  | |
| All-cause mortality | Demographics | Deceased |
|  | ICD-10-CM | R99 |
| MACE – cardiac arrest | ICD-10-CM | I46 |
| MACE – acute myocardial infarction | ICD-10-CM | I21 |
| MACE – cerebral infarction | ICD-10-CM | I63 |
| Organ failure – acute kidney failure | ICD-10-CM | N17 |
| Organ failure – acute respiratory failure | ICD-10-CM | J96.0 |
| Organ failure – ARDS | ICD-10-CM | J80 |
| Organ failure – hepatic failure | ICD-10-CM | K72 |
| Organ failure – disseminated intravascular coagulation | ICD-10-CM | D65 |
| ICU admission | CPT (UMLS) | 1013729 (Critical Care Services) |
| Severe sepsis | ICD-10-CM | R65.2 |
| Covariates for propensity-score matching | | |
| Hypertension | ICD-10-CM | I10 |
| Alcoholic liver disease | ICD-10-CM | K70 |
| Chronic hepatitis | ICD-10-CM | K73 |
| Liver fibrosis/cirrhosis | ICD-10-CM | K74 |
| Other inflammatory liver disease | ICD-10-CM | K75 |
| Ischemic heart disease | ICD-10-CM | I20–I25 |
| Heart failure | ICD-10-CM | I50 |
| Overweight/obesity | ICD-10-CM | E66 |
| Dyslipidemia | ICD-10-CM | E78 |
| Thyroid disorders | ICD-10-CM | E00–E07 |
| Chronic kidney disease | ICD-10-CM | N18 |
| COPD | ICD-10-CM | J44 |
| Dementia | ICD-10-CM | F03 |
| Systemic connective tissue disorders | ICD-10-CM | M30–M36 |
| Malnutrition | ICD-10-CM | E40–E46 |
| Neoplasms (all) | ICD-10-CM | C00–D49 |
| Nicotine dependence | ICD-10-CM | F17 |
| Alcohol-related disorders | ICD-10-CM | F10 |
| Vitamin D deficiency | ICD-10-CM | E55 |
| Hemoglobin | LOINC | 9014 |
| Hemoglobin A1c | LOINC | 9037 |
| Albumin | LOINC | 9045 |
| eGFR (CKD-EPI 2021) | LOINC | 98979-8 |
| AST (GOT) | LOINC | 9047 |
| ALT (GPT) | LOINC | 9044 |
| Total bilirubin | LOINC | 9050 |
| C-reactive protein | LOINC | 9063 |
| Exclusion criteria | | |
| Pregnancy | ICD-10-PCS | 10 |
| Organ transplantation status | ICD-10-CM | Z94 |
| Vitamin B12 supplement use | ATC (NLM) | B03BA |
| Vitamin B12 + folic acid use | ATC (NLM) | B03B |
| Noninfective enteritis/colitis | ICD-10-CM | K50–K52 |
| Toxic liver disease | ICD-10-CM | K71 |
| HIV | ICD-10-CM | B20 |
| Bariatric surgery history | ICD-10-CM | Z98.84 |
| End-stage renal disease | ICD-10-CM | N18.6 |
| CKD stage 4 | ICD-10-CM | N18.4 |
| CKD stage 5 | ICD-10-CM | N18.5 |
| Dialysis dependence | ICD-10-CM | Z99.2 |
| Hematologic malignancies | ICD-10-CM | C81–C96 |
